# Characterisation of patients referred to a tertiary-level inherited cardiac condition clinic with suspected arrhythmogenic right ventricular cardiomyopathy (ARVC)

**DOI:** 10.1186/s12872-022-03021-w

**Published:** 2023-01-12

**Authors:** A. Aljehani, T. Kew, S. Baig, H. Cox, L. C. Sommerfeld, B. Ensam, M. Kalla, R. P. Steeds, L. Fabritz

**Affiliations:** 1 Institute of Cardiovascular Sciences, University of Birmingham, Birmingham, UK; 2 Department of Cardiology, University Hospitals Birmingham NHS Foundation Trust, Birmingham, UK; 3 King Saud Bin Abdulaziz University For Health Sciences, Echocardiography Cardiovascular Technology (ECVT) Program, Riyadh, Saudi Arabia; 4 West Midlands Regional Genetics Unit, Clinical Genetics, Birmingham Women’s and Children’s NHS Foundation Trust (BWC) Birmingham, Birmingham, UK; 5 Department of Cardiology, University Heart and Vascular Centre Hamburg, UKE Hamburg and DZHK, Hamburg/Kiel/Luebeck, Germany; 6 University Centre of Cardiovascular Science, UKE Hamburg, Hamburg, Germany

**Keywords:** Arrhythmogenic right ventricular cardiomyopathy, Arrhythmogenic cardiomyopathy, Electrocardiography, Cardiac magnetic resonance imaging, Echocardiography

## Abstract

**Background:**

Arrhythmogenic right ventricular cardiomyopathy (ARVC) or arrhythmogenic cardiomyopathy is a rare inherited disease with incomplete penetrance and an environmental component. Although a rare disease, ARVC is a common cause of sudden cardiac death in young adults. Data on the different stages of ARVC remains scarce. The purpose of this study is to describe the initial presentation and cardiac phenotype of definite and non-definite ARVC for patients seen at a tertiary service.

**Methods:**

This is a single centre, observational cohort study of patients with definite and non-definite ARVC seen at the Inherited Cardiac Conditions services at University Hospital Birmingham (UHB) in the period 2010–2021. Patients were identified by interrogation of digital health records, medical history, imaging and by examining 12-lead electrocardiograms (ECG).

**Result:**

The records of 1451 patients were reviewed; of those, 165 patients were at risk of ARVC (mean age 41 ± 17 years, 56% male). 60 patients fulfilled task force criteria for definite ARVC diagnosis (n = 40, 67% males), and 38 (72%) of them carried a known pathogenic variant. The remaining 105 patients (50% males) were non-definite, and of these 45 (62%) carried a known pathogenic variant. Patients in the definite group were more symptomatic, with palpitations (57% vs. 17%), syncope (35% vs. 6%) and shortness of breath (28% vs. 5%, *p* < 0.001). T-wave inversion in V1-V3 and epsilon waves were observed only in the definite group. Both PR interval and QRS duration were longer in the definite (170 ± 34 ms and 100 ± 19 ms, *p* < 0.001) compared to (149 ± 25 and 91 ± 14 ms, *p* = 0.005). Patients with definite ARVC had significantly larger RV end diastolic areas and significantly reduced biventricular function (RVEDA = 27 ± 10 cm^2^, RVFAC = 37 ± 11% and EF = 56 ± 12%) compared to the non-definite group (RVEDA = 18 ± 4 cm^2^, RVFAC 49 ± 6% and LVEF 64 ± 7%, *p* < 0.001). Sustained ventricular tachycardia (VT) occurred more frequently in the definite group compared to the non-definite group (27% vs. 2%, *p* < 0.001). Ventricular fibrillation was observed in the definite group only (8 of 60 patients, 13%).

**Conclusion:**

Our study showed differences between definite and non-definite ARVC patients in terms of clinical, electrophysiological and imaging features. Major adverse cardiac events occurred more commonly in the definite group, but also were observed in non-definite ARVC. This single centre observational cohort study forms a basis for further prospective multicentre interventional studies.

**Supplementary Information:**

The online version contains supplementary material available at 10.1186/s12872-022-03021-w.

## Introduction

Arrhythmogenic right ventricular (RV) dysplasia/cardiomyopathy (ARVC) is a rare inherited non-ischemic cardiomyopathy characterised by fibrotic replacement of cardiac myocytes [1]. It is linked to a high incidence of ventricular arrhythmia (VA) and sudden cardiac death (SCD), particularly in young and athletic people [2]. ARVC has an estimated prevalence of 1 in 1000 to 1 in 2000 individuals [3]. Classically, it is considered an inherited disease with an autosomal dominant pattern; nevertheless, incomplete penetrance, variable expressivity and age at manifestation have been described [4]. In patients with classical ARVC, a pathogenic variant in the desmosomal genes *desmoplakin (DSP), plakoglobin (JUP), plakophilin-2 (PKP2), desmocollin-2 (DSC2)* and *desmoglein-2 (DSG2)* accounts for over 50% of cases [5]. Less common pathogenic variants in the extra-desmosomal genes, including *desmin (DES), sodium voltage-gated channel alpha subunit (SCN5A), phospholamban (PLN), transmembrane protein 43 (TMEM43), N-cadherin (CDH2) and alpha-T-catenin (CTNNA3),* have also been reported [6].

The first clinical description of ARVC by Marcus et al. [7] noted a series of 24 patients presenting with T-wave inversion in the right precordial electrocardiogram (ECG) leads, dilatation of the right ventricle (RV) and dysplasia associated with ventricular tachycardia (VT) of left bundle branch block configuration. In 1994, the first ARVC Task Force criteria (TFC) for diagnosis based on histological, arrhythmia, structural, ECG and familial features of the disease were published. In 2010, these criteria were modified to include quantitative parameters, particularly from imaging [8], leading to increased specificity for the diagnosis. However, sensitivity was still lacking, particularly for early stages of the disease [9]. New insights from post-mortem studies, genotype–phenotype correlation studies and myocardial tissue characterisation by cardiovascular magnetic resonance imaging (CMR) have led to a broader understanding of the disease. Recent modifications have been made to the classification of ARVC as ‘arrhythmogenic cardiomyopathy’, with recognition of biventricular and predominant left ventricular phenotypes. Because of the rarity of the condition and sudden death as a frequent first manifestation, phenotypic data on cohorts referred for assessment for ARVC are still scarce. Therefore, the purpose of this study is to describe the initial presentation and natural history of patients with ARVC referred to a tertiary care centre and to investigate the difference in cardiac phenotypes in definite and non-definite ARVC.

## Methods

### Study design

**Fig. 1:**
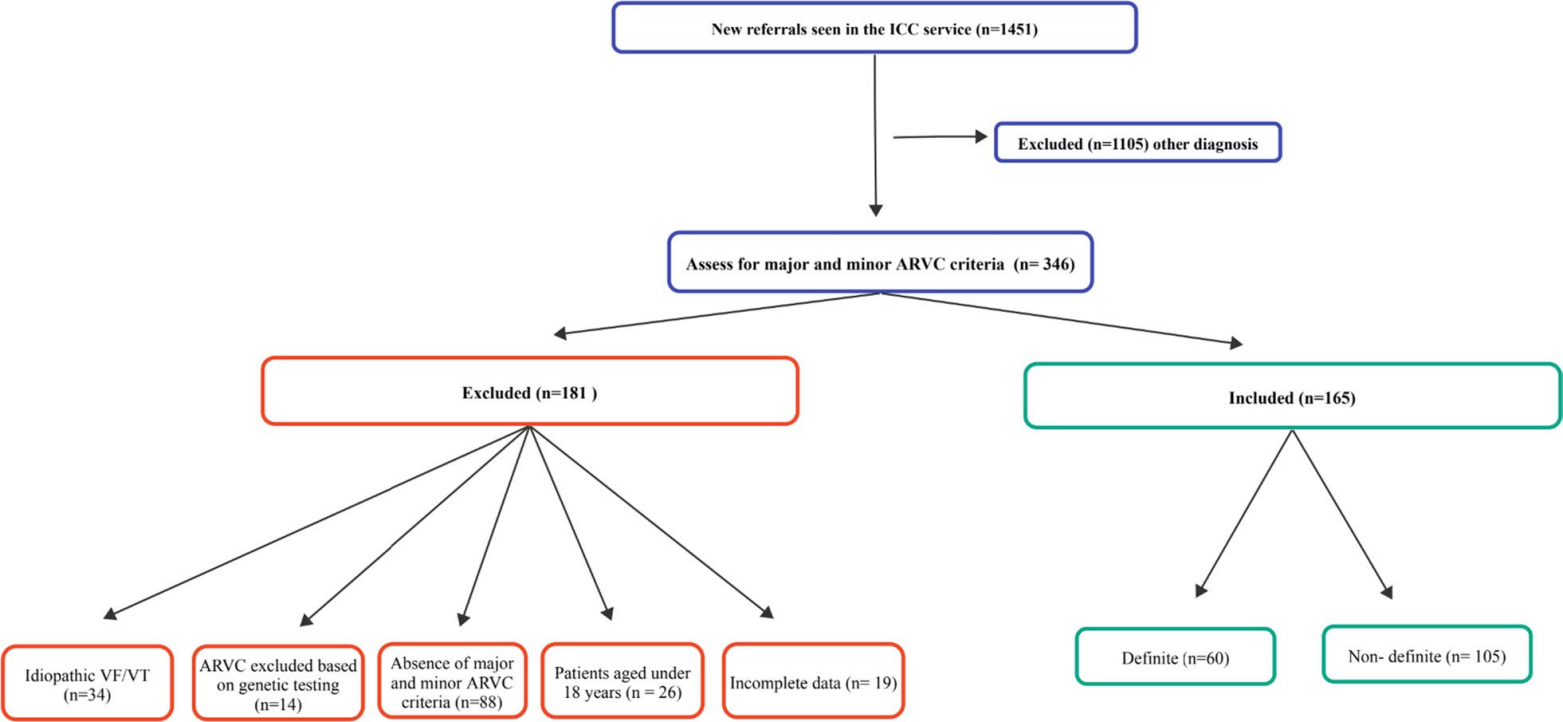
Patients referred to UHB with proven or suspected arrhythmia syndrome

This is a single centre, observational, cross-sectional cohort study conducted at the Inherited Cardiac Conditions (ICC) service at University Hospital Birmingham (UHB). This is a highly specialised clinic to which patients with a suspected inherited cardiac condition are referred, often following aborted or sudden cardiac death either in the individual or in a close relative (Fig. 1). The scope of the clinic was set up by the NHS Commissioning Board to include the following four disease categories: (1) arrhythmia syndromes caused by variants in the proteins involved in generating action potential, for example, Brugada syndrome, long QT syndrome and short QT syndrome; (2) cardiomyopathies caused mainly by variants in the proteins involved in myocardial contraction, for example, hypertrophic cardiomyopathy, dilated cardiomyopathy and ARVC; (3) inherited arteriopathies, for example, Loeys–Dietz syndrome and Marfan syndrome; and (4) muscular dystrophies. The ICC clinic at UHB focuses on arrhythmia syndromes and cardiomyopathies with a joint board, with additional hypertrophic cardiomyopathy clinics and with separate arteriopathy and muscular dystrophy services. The aim of the service is to provide timely diagnosis, proactive care and a smooth transition from paediatric to adult care. The ICC functions as a multidisciplinary service, with a core consisting of cardiologists, clinical geneticists and counsellors, ICC nurse specialists and experienced echocardiographers. Diagnostic tools include echocardiography, CMR, exercise testing, signal-averaged ECGs (SAECG), and genetic testing.

### Study population

Adult patients aged 18 and older were included consecutively based on referral to the clinic with suspected ARVC or a positive family history [10]. Patients were excluded if they had a history or current evidence of ischaemia, hypertension or valve disease as the most likely cause of heart disease and cardiovascular complication (Table 1). Final diagnostic categorisation into definite and non-definite categories was carried out following discussion by a multidisciplinary team with the ICC clinicians, including specialists in genetics, cardiac electrophysiology and cardiovascular imaging all experienced in ARVC. All patients with suspected and confirmed ARVC were identified by interrogation of digital health records and were classified into two groups, ‘definite’ and ‘non-definite according to the 2010 TFC [8]. A definite ARVC diagnosis was made when two major criteria or one major and two minor criteria or four minor criteria from different categories were met. A non-definite ARVC diagnosis was made when one major and one minor criteria or three minor criteria from different categories were met or when one major or two minor criteria from different categories were met. Major adverse events were recorded from first attendance and medication, biomarkers and SAECG were included as close to the first attendance as feasible from the clinical records.

**Table 1:** Inclusion and exclusion criteria of the study group

*Inclusion criteria*
Patients > 18 years
Patient with definite ARVC diagnosis
Patients with non-definite ARVC diagnosis (one major and one minor criteria or three minor criteria from different categories or one major or two minor criteria from different categories)
*Exclusion criteria*
Patients < 18 years
Patients with history of ischaemia
Patients with history of hypertension
Patients with history of valve disease

As exercise has been described as an aggravating factor for ARVC, proportions of different levels of physical activity (competitive and non-competitive exercise) were compared between definite and non-definite groups, as defined by Maron et al. [11].

### Electrocardiography

12-lead ECGs were performed in all cases upon entry to the ICC clinic. Each 12-lead ECG was assessed for the presence of repolarisation and depolarisation criteria, per the 2010 TFC. Signal-averaged ECGs were performed routinely at baseline to assess the presence or absence of late potentials. Premature ventricular complex count was measured by 24-h Holter monitoring upon entry to the ICC clinic and was considered abnormal if it was > 500/24 h, as described by 2010 TFC.

### Echocardiography

Transthoracic echocardiography (TTE) was performed by experienced echocardiographers accredited by the British Society of Echocardiography (BSE). All available TTE images were re-analysed and re-measured offline by a single independent operator (AA) blinded to demographic, clinical and diagnostic data for conventional parameters, including atrial and ventricular size and function per the most recent requirements of the published BSE guidelines using IntelliSpace Cardiovascular technology (ISCV; Philips, the Netherlands) [12].

### Cardiac magnetic resonance imaging

Left and right ventricular volumes and mass and left and right atrial volumes were acquired in line with standard CMR (1.5 T Avanto; Siemens Healthcare, Erlangen, Germany) protocols [13]. Late gadolinium enhancement (LGE) imaging was performed 7–10 min after 0.15 mmol/kg of gadolinium-based contrast agent administration (Gadovist Bayer Healthcare). Analysis of the right and left ventricular volume and ejection fraction were performed using cvi42® (version 5.3.6 Circle Cardiovascular Imaging, Canada) by a single independent operator (AA) blinded to the demographic, clinical and diagnostic data for conventional parameters. For ventricular volume analysis, the endocardial border was detected with the largest and smallest cavity volumes defined as end diastole and end systole, respectively. The endocardial border was defined as the boundary between blood pool and myocardium, with papillary muscles excluded from ventricular volume.

### Genetics

Genetic testing was performed with either next-generation sequencing or whole genome targeted panel. The panel of genes included *Plakophilin-2 (PKP2), desmoglein-2 (DSG2) plakoglobin (JUP), desmoplakin (DSP), desmocollin-2 (DSC2), N-cadherin (CDH2), phospholamban (PLN), transmembrane protein 43 (TMEM43), desmin (DES) and lamin A (LMNA).* Genetic testing was performed for family members only after identification of a pathogenic variant in the proband and in clinically screened family members of a deceased proband who expressed an ARVC phenotype.

### Statistical analysis

Continuous variables are presented as means and standard deviations; the unpaired t-test was used for statistical comparison between definite and non-definite ARVC groups. Additionally, categorical variables are expressed as a frequency with percentage of the total population, having been compared statistically using a Fisher’s exact test or a Chi-squared test. For non-normally distributed variables, a Mann–Whitney U Test was used. A two-sided p value with statistical significance defined as < 0.05 was used. All statistical tests were performed using IBM SPSS 27.

## Results

A total of 1451 new patients were referred to the ICC clinic in the period 2010–2021 for confirmed or suspected arrhythmia syndromes and differential diagnosis of ARVC. Of those, 1105 patients were excluded with another diagnosis; the most common categories were the following: long QT syndrome: n = 355; hypertrophic cardiomyopathy (HCM): n = 338; dilated cardiomyopathy (DCM): n = 140; Brugada syndrome: n = 121 and catecholaminergic polymorphic ventricular tachycardia (CPVT): n = 31.

There were also disparate other conditions diagnosed in this subspecialised clinic which were seen in low numbers (as these patients were more often diagnosed and seen in other highly specialised tertiary care clinics), including: peripartum cardiomyopathy: n = 3; restrictive cardiomyopathy: n = 1; LV non-compaction cardiomyopathy: n = 7; congenital heart disease: n = 10; Fabry disease: n = 1; Wolf Parkinson White syndrome (WPWS): n = 5, coronary artery disease: n = 10; familial hypercholesterolemia: n = 2; familial lone AF: n = 7; family history of SCD due to known aetiology-other than ARVC: n = 41 and patients or family members below age 18: n = 33).

346 patients were assessed for the presence of major and minor ARVC criteria as per the 2010 TFC, and 181 patients were excluded (those patients who did not meet either definite or non-definite ARVC diagnosis n = 28; ARVC excluded based on genetic testing that was negative for the family member of a proband with confirmed pathogenic variant: n = 14; patients aged under 18 years screened for ARVC: n = 26; incomplete data: n = 19; Idiopathic VF/VT: n = 34; and family history of SCD due to unknown aetiology with normal investigations: n = 60. A definite diagnosis of ARVC was made for 60 patients, and non-definite disease was diagnosed in 105 patients (Fig. 1).

### Clinical characteristics

Overall, average age at presentation was 41 ± 17 years, and 56% of patients were male (n = 92). The majority of those referred to the clinic following a possible or confirmed diagnosis of ARVC in a first-degree relative (n = 118 (72%)) or a second-degree relative (n = 21 (13%)). Some patients were diagnosed ‘de novo’ following a major adverse cardiovascular event, including sustained VT (n = 18 (11%)) and VF (n = 8 (5%)). The most common presentations were palpitations (n = 52 (32%)) and syncope (n = 27 (16%)). Of the total population, 29 (19%) patients had a history of competitive exercise.

The baseline demographic characteristics of those with ARVC are summarised in Table 2, sub-divided according to definite and non-definite ARVC diagnosis. Patients in the definite group were more symptomatic compared to those in the non-definite, with palpitations (57% vs. 17%), syncope (35% vs. 6%) and shortness of breath (28% vs. 5%, *p* < 0.001). History of participation in competitive sports was more common in the definite group (32%) compared to the non-definite group (11%, *p* = 0.001). Major adverse cardiac events were more common in the definite group compared to the non-definite group in terms of aborted VF (13% vs. 0%) and sustained VT (27% vs. 2%, *p* < 0.001).

**Table 2:** Clinical and demographic biomarkers of the study cohort

Clinical characteristics	Total	Definite n = 60	non-definite n = 105	*P* value
Age, mean ± SD	41 ± 17	44 ± 16	40 ± 18	0.086
Male gender, n (%)	92 (56)	40 (67)	52 (50)	0.036
*Ethnicity*				
White, n (%)	89 (54)	36 (22)	53 (32)	–
Asian, n (%)	22 (13)	10 (6)	12 (7)	–
Mixed, n (%)	2 (1)	0 (0)	2 (1)	–
Black, n (%)	6 (4)	5 (3)	1 (1)	–
Not specified, n (%)	46 (28)	9 (5)	37 (22)	–
Weight, mean ± SD	79 ± 21	81 ± 23	78 ± 20	0.465
Height, mean ± SD	169 ± 16	169 ± 18	169 ± 15	0.936
BSA, mean ± SD	2 ± 1.4	2.2 ± 2.3	1.9 ± 0.2	0.284
^a^*History of sport*				
History of competitive sport, n (%)	29 (19)	19 (32)	10 (11)	0.001
History of non-competitive sport, n (%)	31 (20)	11 (19)	20 (22)	0.837
Not performing any types of sport n (%)	92 (61)	29 (49)	63 (68)	0.027
*Symptoms*				
Palpitation, n (%)	52 (32)	34 (57)	18 (17)	< 0.001
Syncope, n (%)	27 (16)	21 (35)	6 (6)	< 0.001
Shortness of breath, n (%)	22 (13)	17 (28)	5 (5)	< 0.001
*Family history*				
Major ARVC confirmed in FDR by clinical or pathogenic (autopsy or surgical) criteria n (%)	104 (63)	24 (40)	80 (76)	< 0.001
^b^Major identification of pathogenic variant associated with ARVC in the pts under evaluation n (%)	83 (66)	38 (72)	45 (62)	0.260
Minor history of ARVC in FDR relative but they cannot be confirmed premature (SCD < 35) due to suspect with ARVC in FDR n (%)	14 (8)	5 (8)	9 (9)	1.000
Minor ARVC confirmed by clinical or pathogenic (autopsy or surgical criteria in SDR n (%))	21 (13)	7 (12)	14 (13)	0.813
*Medication*				
Statins, n (%)	5 (3)	3 (5)	2 (2)	0.355
Anticoagulant, n (%)	8 (5)	6 (10)	2 (2)	0.028
Antiarrhythmic drugs, n (%)	9 (5)	8 (13)	1 (1)	0.001
Beta blocker, n (%)	21 (13)	19 (32)	2 (2)	< 0.001
*Major adverse cardiac events*				
ICD implanted, n (%)	12 (7)	11 (18)	1 (1)	< 0.001
Ventricular fibrillation (VF), n (%)	8 (5)	8 (13)	0 (0)	< 0.001
Sustained ventricular tachycardia (VT), n (%)	18 (11)	16 (27)	2 (2)	< 0.001
Heart failure (HF), n (%)	3 (2)	3 (5)	0 (0)	0.047
Atrial fibrillation/flutter, n (%)	7 (4)	5 (8)	2 (2)	0.100
*Biomarkers*				
NT-proBNP (ng/L), median, (IQR)	299 (45- 498)	450 (265- 740)	38 (29- 96)	< 0.001
Na (mmol/L), median, (IQR)	140 (138–141)	140 (70–101)	140 (138–141)	0.661
K (mmol/L), median, (IQR)	4.3 (4—4.6)	4.4 (4.1—4.8)	4.2 (3.9–4.4)	0.065
Creatinine (µmol/L), median, (IQR)	77 (67–92)	81 (138–142)	74 (62—85)	0.071

BSA: body surface area; FDR: first degree relative; SDR: second degree relative; ICD: implantable cardioverter-defibrillator; NT-proBNP: B-type natriuretic peptide; Na: sodium; K: potassium;

^a^Information on history of performed sports was available for 152 patients only

^b^Genetic testing was performed for 127 patients only

### Electrocardiographic evaluation

12-lead ECG, SAECG and 24-h Holter data from the definite and non-definite ARVC groups are summarised in Table 3. T-wave inversion (TWI) in leads V1–V3 detected by 12-lead ECG was observed in 36 (60%) patients in the definite group. Epsilon waves were observed only in the definite group (n = 14, 23%), while no patient in the non-definite group demonstrated a major ECG depolarisation criterion (*p* < 0.001). There were no significant differences between the definite and non-definite groups regarding the 12-lead ECGs in terms of P and QRS axes, but both PR interval and QRS duration were longer in the definite group (170 ± 34 ms and 100 ± 19 ms) compared to non-definite group (149 ± 25, *p* < 0.001 and 91 ± 14 ms *p* = 0.005).). Patients in the definite group were more likely to have BBB compared to those in the non-definite (23% vs. 5%, *p* < 0.001).

**Table 3:** Electrical characteristics of definite and non-definite ARVC

Parameter	Total	Definite n = 60	non-definite n = 105		*P* value
*Depolarisation criteria*					
Major criteria, n (%), Epsilon wave in the right precordial leads (V1–V3)	14 (8)	14 (23)	0 (0)		< 0.001
^a^Minor criteria, n(%) Signal-averaged ECG with late potential (if QRS on standard surface < 110 ms)	71 (52)	33 (70)	38 (42)		0.002
*Repolarisation criteria*					
Major criteria, n (%), TWI in right precordial leads (V1, V2 and V3)	36 (22)	36 (60)	0 (0)		< 0.001
Any minor criteria, n (%), TWI in leads V1 and V2 or in V4, V5, and V6, TWI in leads V1, V2, V3, and V4 with RBBB	32 (19)	27 (45)	5 (5)		< 0.001
BBB, n (%)	19 (12)	14 (23)	5 (5)		< 0.001
^b^> 500 PVC / 24 h (Holter), n (%)	23 (37)	20 (67)	3 (9)		< 0.001
Heart rate (Bpm)	67 ± 14	64 ± 15	69 ± 13		0.018
PR interval (ms)	157 ± 30	170 ± 34	149 ± 25		< 0.001
QRS duration (ms)	94 ± 16	100 ± 19	91 ± 14		0.005
QT (ms)	403 ± 39	421 ± 41	393 ± 33		< 0.001
QTc (ms)	419 ± 28	425 ± 27	415 ± 28		0.024
P axes	49 ± 26	53 ± 17	47 ± 29		0.107
QRS axes	38 ± 41	31 ± 51	42 ± 34		0.122
T axes	31 ± 37	20 ± 43	37 ± 32		0.010
*Signal-averaged ECG (SAECG)*					
Total QRS duration (filtered) (ms)		116 ± 26	128 ± 32	111 ± 21	0.002
Duration of HFLA signals < 40 mv (ms)		40 ± 25	52 ± 30	34 ± 20	< 0.001
RMS voltage in terminal 40 ms (mV)		34 ± 26	23 ± 18	40 ± 28	< 0.001
Mean voltage in terminal 40 ms (mV)		24 ± 20	17 ± 15	27 ± 21	0.001

TWI:T wave inversion; RBB: right bundle branch block; BBB: bundle branch block; PVC: premature ventricular contractions; HFLA: high frequency low amplitude; RMS: root mean square

^a^SAECG was performed in 137 patients

^b^24 Holter monitoring was performed in 63 patients

### Imaging evaluation

**Fig. 2:**
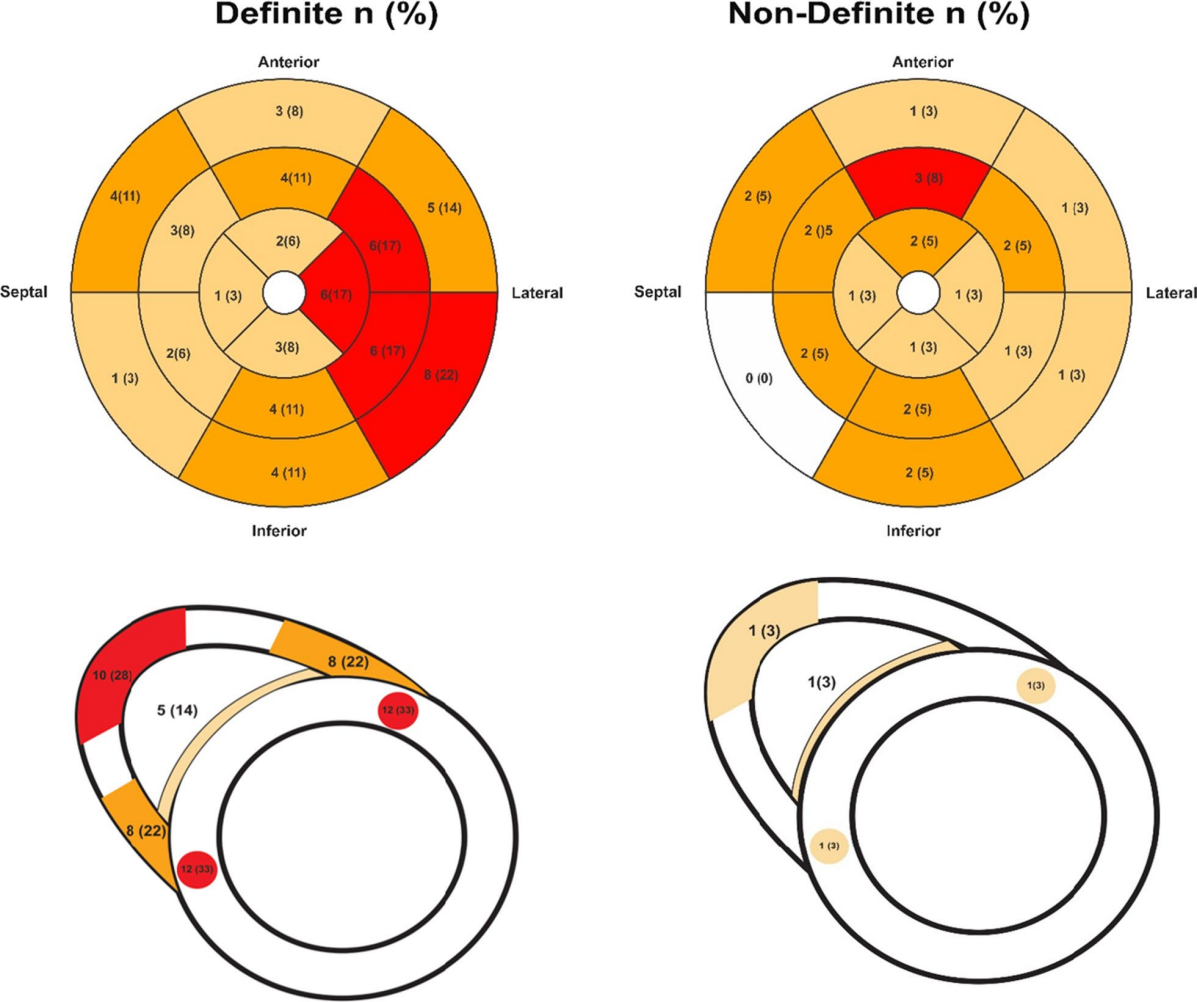
Left ventricular (LV) and right ventricular (RV) LGE distribution on CMR in definite and non-definite ARVC patients

Imaging characteristics derived from echocardiography and CMR are summarised in Table 4. At presentation, RV function in the group with definite ARVC was worse by fractional area change (FAC) on echocardiography compared to the non-definite ARVC group (37 ± 11% vs. 49 ± 6%, *p* < 0.001) and right ventricular ejection fraction (RVEF) on CMR (39 ± 15% vs. 56 ± 7%, *p* < 0.001). Likewise, RV size was larger in the definite group compared to the non-definite group based on both imaging modality. Late enhancement was more common in those with definite ARVC compared to those with non-definite ARVC, although detectable in 5 (13%) of the patients in the non-definite ARVC group (*p* < 0.001), (Fig. 2). (Additional file 1: Figs. S1, S2 and S3) illustrate imaging and ECG features taken from one patient with definite and one patient with non-definite ARVC.

**Table 4:** Imaging-derived characteristics of definite and non-definite ARVC

Parameters	Total n = 165	Definite n = 60	non-definite n = 105	*P* value
*Echocardiography*				
RA area (cm^2^)	16 ± 6	19 ± 8	14 ± 4	< 0.001
*RV data*				
RVOT PLAX (cm)	3.0 ± 0.8	3.5 ± 0.9	2.7 ± 0.5	< 0.001
Proximal RVOT PSAX (cm)	3.2 ± 0.7	3.6 ± 0.8	3.0 ± 0.6	< 0.001
4C base RV1(cm)	3.6 ± 0.8	4.2 ± 1.0	3.3 ± 0.6	< 0.001
4C mid RV2 (cm)	3.1 ± 0.7	3.5 ± 0.9	2.9 ± 0.5	< 0.001
4C length RV3 (cm)	7.0 ± 1.1	7.5 ± 1.3	6.7 ± 0.9	< 0.001
RVEDA (cm^2^)	21 ± 8	27 ± 10	18 ± 4	< 0.001
RVESA (cm^2^)	12 ± 7	17 ± 8	9 ± 3	< 0.001
FAC (%)	44 ± 10	37 ± 11	49 ± 6	< 0.001
TAPSE (cm)	2.2 ± 0.5	1.9 ± 0.5	2.3 ± 0.4	< 0.001
*LV data*				
EDV (ml)	91 ± 29	89 ± 27	93 ± 31	0.443
ESV (ml)	36 ± 15	39 ± 16	33 ± 14	0.037
EF (%)	61 ± 10	56 ± 12	64 ± 7	< 0.001
LA area (cm^2^)	16 ± 4	17 ± 4	15 ± 3	0.023
LA volume (ml)	39 ± 14	42 ± 17	37 ± 12	0.074
*CMR*				
*RV characteristics*				
RVEDV (ml)	182 ± 55	195 ± 65	168 ± 41	0.032
RVESV (ml)	100 ± 54	125 ± 65	75 ± 20	< 0.001
RV EF (%)	48 ± 14	39 ± 15	56 ± 7	< 0.001
*LV characteristics*				
LVEDV (ml)	155 ± 50	155 ± 60	155 ± 38	0.978
LVESV (ml)	63.9 ± 42	71 ± 55	57 ± 21	0.122
LV EF (%)	61 ± 13	58 ± 17	64 ± 8	0.043
^a^LGE present, n (%)	29 (39)	24 (67)	5 (13)	< 0.001
LV LGE	18 (24)	14 (39)	4 (10)	0.006
RV LGE	25 (33)	22 (61)	3 (8)	< 0.001

RA area: right atrial area; RVOT PLAX: right ventricular outflow tract parasternal long axis; RVOT PSAX: right ventricular outflow tract parasternal short axis; 4C base RV1: 4 chamber right ventricular basal diameter; 4C mid RV2: 4 chamber right ventricular mid diameter; 4C length RV3: 4 chamber right ventricular length; RVEDA: right ventricular end diastolic area; RVESA: right ventricular end systolic area; RVFAC: right ventricular fractional area change; TAPSE: tricuspid annular plane systolic excursion; LVEDD: left ventricular end-diastolic volume; LVESV: left ventricular end-systolic volume; EF: ejection fraction; LA area: left atrial area; LA volume: left atrial volume; LGE: late gadolinium enhancement

^a^LGE was assessed in 75 patients only

## Genetics

Genetic data are summarised in Table 5. Genetic testing was performed in 127 patients out of 165, and a pathogenic variant was identified in 83 (66%). The most common pathogenic variant was PKP2, which occurred in 50 (39%) patients, followed by DSP in 26 (20%) patients. Of those, 7 (6%) patients showed a double variant along with PKP2 and DSP. Around 4 (7%) patients with double variants were in the definite ARVC stage, while 3 (4%) were in the non-definite ARVC stage.

**Table 5:** Genetic testing results for definite and non-definite ARVC

Genetic testing, n (%)	Total n = 165	Definite n = 60	non-definite n = 105	P value
No pathogenic variant identified (NPGVI), n (%)	41 (32)	14 (26)	27 (37)	0.250
Plakophillin-2 (PKP2), n (%)	50 (39)	27 (50)	23 (32)	0.044
Desmoplakin (DSP), n (%)	26 (20)	6 (11)	20 (27)	0.027
Desmocollin-2 (DSC2), n (%)	3 (2)	3 (6)	0 (0)	0.074
Desmoglein-2 (DSG2), n (%)	4 (3)	2 (4)	2 (3)	1.000
Variant of unknown significance (VUS), n (%)	2 (2)	1 (2)	1 (1)	1.000
PKP2 and VUS in DSP	4 (3)	3 (6)	1 (1)	0.311
PKP2 and VUS in TMEM43 (transmembrane protein 43)	1 (1)	1 (2)	0 (0)	0.425
PKP2 and VUS in DSC2	1 (1)	0 (0)	1 (1)	1.000
DSP and VUS in PKP2, n%)	1 (1)	0 (0)	1 (1)	1.000
Awaiting genetic results n (%)	1 (1)	1 (2)	0 (0)	0.425
Not tested, n (%)	38 (23)	6 (10)	32 (30)	0.004

^**a**^Genetic testing was performed in 127 patients only

No known pathogenic variant was detected in 41 (32%) of the patients. DSC2 variants were detected in 3 (2%) patients and DSG2 variants in 4 (3%). Patients in the non-definite group were more likely to harbour a DSP variant (*p* = 0.027).

As 32 (30) patients did not undergo genetic testing we repeated the analysis after excluding the untested patients from the non-definite group and the results presented in the supplementary did not show significant differences from the result presented in the manuscript (Additional file 1: Table S1, S2 and S3).

## Discussion

This is a single centre snapshot of the clinical presentation, demographic, electrical, imaging and genetic characteristics of a cohort of patients referred for ICC assessment for suspected ARVC in the West Midlands region of the United Kingdom. In contrast to prior studies that have mostly focused on the definite group alone, a particular attribute of this study is to present characteristics of both patients with definite ARVC and patients with non-definite ARVC.

Among 165 subjects hitherto screened, a definite ARVC diagnosis was made according to the 2010 TFC for 60 patients and a non-definite ARVC diagnosis was made for 105 patients. As would be expected from a predominant autosomal inherited disease, there was near equivalence in terms of sex in the whole population described here (56% male), but there was a male preponderance in the definite group (67% male). In the original paper by Marcus et al., a male to female ratio of 2.7:1 was described in a study of 24 patients [7]. Likewise, in a study of 149 index patients, there was significant male predominance in both ARVC patients with a confirmed pathogenetic variant (71% male) and those without (80% male), [14]. This finding of male predominance is not to be expected based on the inheritance of this condition. It could however be caused by a more active lifestyle, as 9 (23%) males reported high intensity sports-physical, and by male sex hormone facilitating disease phenotype [15]. The higher diagnosis rate of definite ARVC in males could also reflect a lack of sensitivity of the imaging criteria, even when indexed to body surface area for women. Echocardiography cut-off values are based not only on absolute values but also values indexed to body surface area for size that are the same in men and women; yet, even indexed dimensions are frequently smaller in women.

Among individuals with ARVC, a history of exercise has been linked to disease progression and poor outcomes. Variant carriers who exercised for longer periods of time were previously shown to have a higher risk of VA events [16]. 32% of our definite group exercised regularly and had suffered more potentially life-threatening arrhythmias at presentation compared to the non-definite ARVC group. This is consistent with lower penetrance in patients who exercise less. In patients with definite ARVC, the same result was found; athletes with confirmed ARVC had a greater risk of VA at a younger age and more often received an implantable cardioverter-defibrillator [17]. Although patients in the definite ARVC group were more likely to experience severe arrhythmia and to show structural involvement, two patients (2%) in the non-definite group experienced sustained VT. This suggests that severe arrhythmia may occur at any stage of the disease, even in the absence of myocardial structural changes, making risk stratification challenging for those patients.

5 patients (13%) with non-definite ARVC diagnosis were found to have LGE in one or both ventricles (Table 4) (Fig. 2). Of those, three patients showed enhancement in the mid-anterior wall of the LV. In line with this, it has been reported that the subtricuspid/peritricuspid area and the LV inferolateral wall may be the most affected areas in the early stages of ARVC, and that the finding of LGE in ARVC patients is a predictor of all cause mortality and major adverse cardiovascular events [18]. Although LGE was not included in the 2010 TFC, our finding suggests the diagnostic utility of CMR-LGE to define those with non-definite ARVC disease.

Genetic family screening identifies individuals at risk of developing ARVC [19]. However, the current standard methods for screening do not identify all non-definite patients at risk of severe arrhythmia and may miss early structural involvement. Newer imaging techniques, such as deformation imaging by echocardiography, CMR-LGE and exercise echocardiography, could detect early structural involvement and identify those patients at risk, as several studies suggest. A study conducted by Leren et al. [19] aimed at investigating early arrhythmic markers in early ARVC. Of 73 patients with early ARVC, arrhythmic events occurred in 15 (21%). Those with arrhythmic events showed longer RV mechanical dispersion (RVMD), larger RV basal diameter and abnormal SAECG. These findings were replicated, with shorter RVMD in asymptomatic variant carriers compared with ARVC patients with arrhythmia. Importantly, biventricular strain was reduced, and MD was more pronounced in asymptomatic variant carriers compared to controls [20].

### Limitations

Despite the fact that participants were recruited from a regional institution servicing a varied multi-ethnic community in the Midlands, the data were acquired from a single centre, which may restrict the generalisability of our findings. As not all our non-definite ARVC patients underwent genetic testing, our results could be confounded by the inclusion of untested first-degree relatives who might not have true ARVC. Larger non-definite ARVC cohorts are needed to confirm our results. Finally, cardiac magnetic resonance, SAECG, 24-h Holter monitoring and blood tests were not performed for all patients.

## Conclusion

The findings of this study showed differences in clinical, electrical and imaging phenotype between definite and non-definite ARVC patients. Major adverse events were more common in definite ARVC patients but also occurred in non-definite ARVC. This single centre observational cohort study forms a basis for further prospective multicentre interventional studies.

## Supplementary Information

**Additional file 1**. Represents clinical, electrical and imaging data after excludig untested non-definite ARVC patients.

## Data Availability

The anonymised datasets used and/or analysed during the current study are available from the corresponding author upon reasonable request.

## Abbreviations

ARVC: Arrhythmogenic right ventricular cardiomyopathy
VA: Ventricular arrhythmias
SCD: Sudden cardiac death
CMR: Cardiovascular magnetic resonance
ECG: Electrocardiogram
ICD: Implantable cardioverter-defibrillator
LGE: Late gadolinium enhancement
LV: Left ventricle
RV: Right ventricle
SCD: Sudden cardiac death
TFC: Task force criteria
