# Supplementary material for: Characterisation of patients referred to a tertiary-level inherited cardiac condition clinic with suspected arrhythmogenic right ventricular cardiomyopathy (ARVC)
Source: BMC Cardiovasc Disord. 2023 Jan 12;23:14. doi: 10.1186/s12872-022-03021-w (PMC9837886; doi:10.1186/s12872-022-03021-w)
Supplement: Supplementary file 1 — Additional file 1. Represents clinical, electrical and imaging data after excludig untested non-definite ARVC patients. [file 12872_2022_3021_MOESM1_ESM.docx]

**Supplementary**

**After excluding untested non-definite ARVC patients, n=32 (30%).**

**eTable 1.** Clinical and demographic biomarkers of the study cohort

| **Clinical characteristics** | **Total, n=133** | **Definite**  **n = 60** | **Non-definite n=** **73** | **P value** |
| --- | --- | --- | --- | --- |
| Age, mean ± SD | 43 ± 17 | 44 ± 16 | 41 ± 18 | 0.282 |
| Male gender, n (%) | 78 (59) | 40 (67) | 38 (52) | 0.112 |
| Weight, mean ± SD | 79 ± 22 | 81 ± 23 | 77 ± 21 | 0.336 |
| Height, mean ± SD | 169 ± 17 | 169 ± 18 | 168 ± 16 | 0.709 |
| BSA, mean ± SD | 2.0 ± 1.5 | 2.2 ± 2.3 | 1.9 ± 0.2 | 0.260 |
| ^a^ History of sport | | | | |
| History of competitive sport, n (%) | 27 (22) | 19 (32) | 8 (13) | 0.10 |
| History of non-competitive sport, n (%) | 25 (20) | 11 (19) | 14 (22) | 0.823 |
| Not performing any types of sport n (%) | 71 (58) | 29 (49) | 42 (66) | 0.071 |
| **Symptoms** | | | | |
| Palpitation, n (%) | 48 (36) | 34 (57) | 14 (19) | < 0.001 |
| Syncope, n (%) | 25 (19) | 21 (35) | 4 (5) | < 0.001 |
| Shortness of breath, n (%) | 20 (15) | 17 (28) | 3 (4) | < 0.001 |
| **Family history** | | | |  |
| Major ARVC confirmed in FDR by clinical or pathogenic (autopsy or surgical) criteria n (%) | 74 (56) | 24 (40) | 50 (68) | 0.002 |
| ^b^ Major identification of pathogenic variant associated with ARVC in the pts under evaluation n (%) | 83 (66) | 38 (72) | 45 (62) | 0.260 |
| Minor history of ARVC in FDR relative but they cannot be confirmed premature (SCD < 35) due to suspect with ARVC in FDR n (%) | 12 (9) | 5 (8) | 7 (10) | 1.000 |
| Minor ARVC confirmed by clinical or pathogenic (autopsy or surgical criteria in SDR n (%)) | 21 (16) | 7 (12) | 14 (19) | 0.340 |
| **Medication** | | | | |
| Statins, n (%) | 4 (3) | 3 (5) | 1 (1) | 0.327 |
| Anticoagulant, n (%) | 7 (5) | 6 (10) | 1 (1) | 0.046 |
| Antiarrhythmic drugs, n (%) | 9 (7) | 8 (13) | 1 (1) | 0.011 |
| Beta blocker, n (%) | 21 (16) | 19 (32) | 2 (3) | < 0.001 |
| **Major adverse cardiac events** | | | | |
| ICD implanted, n (%) | 12 (9) | 11 (18) | 1 (1) | 0.001 |
| Ventricular fibrillation (VF), n (%) | 8 (6) | 8 (13) | 0 (0) | 0.001 |
| Sustained ventricular tachycardia (VT), n (%) | 18 (14) | 16 (27) | 2 (3) | < 0.001 |
| Heart failure (HF), n (%) | 3 (2) | 3 (5) | 0 (0) | 0.089 |
| **Biomarkers** | | | | |
| NT-proBNP (ng/L), median, (IQR) | 395 (169-618) | 450 (265- 740) | 58 (39 -170) | < 0.001 |
| Na (mmol/L), median, (IQR) | 140 (138 -142 ) | 140 (70 -101) | 140 (139 - 141) | 0.691 |
| K (mmol/L), median, (IQR) | 4.4 (4.1- 4.6) | 4.4 (4.1 - 4.8) | 4.3 (4.1- 4.5) | 0.281 |
| Creatinine (µmol/L), median, (IQR) | 78 (68-98) | 81 (138 – 142) | 75 (64 -92) | 0.219 |

*Abbreviations : BSA: body surface area; FDR: first degree relative; SDR: second degree relative; ICD: implantable cardioverter-defibrillator; NT-proBNP: B-type natriuretic peptide; Na: sodium; K: potassium ^a^ Information on history of performed sports was available for 123 patients only.*

*^b^ Genetic testing was performed in 127patients only.*

**eTable 2.** Electrical characteristics of definite and **Non-definite** ARVC

| **Parameter** | **Total, n=133** | **Definite**  **n = 60** | **Non-definite n=** **73** | | **P value** |
| --- | --- | --- | --- | --- | --- |
| **Depolarisation criteria** | | | | | |
| Major criteria, n (%), Epsilon wave in the right precordial leads (V1–V3) | 14 (11) | 14 (23) | 0 (0) | | < 0.001 |
| ^a^ Minor criteria, n (%) Signal-averaged ECG with late potential (if QRS on standard surface <110 ms) | 58 (53) | 33 (70) | 25 (40) | | 0.002 |
| **Repolarisation criteria** | | | | | |
| Major criteria, n (%), TWI in right precordial leads (V1, V2 and V3) | 36 (27) | 36 (60) | 0 (0) | | < 0.001 |
| Any minor criteria, n (%), TWI in leads V1 and V2 or in V4, V5, and V6, TWI in leads V1, V2, V3, and V4 with RBBB | 30 (23) | 27 (45) | 3 (4) | | < 0.001 |
| BBB, n (%) | 18 (14) | 14 (23) | 4 (6) | | 0.004 |
| ^b^ >500 PVC / 24 hours (Holter), n (%) | 22 (44) | 20 (67) | 2 (10) | | < 0.001 |
| Heart rate (Bpm) | 66 ± 13 | 64 ±15 | 68 ±12 | | 0.089 |
| PR interval (ms) | 160 ± 30 | 170 ± 34 | 153 ± 24 | | 0.002 |
| QRS duration (ms) | 96 ± 16 | 100 ± 19 | 93 ±12 | | 0.015 |
| QT (ms) | 408 ± 39 | 421 ± 41 | 398 ± 34 | | <0.001 |
| QTc (ms) | 420 ± 28 | 425 ± 27 | 416 ± 28 | | 0.051 |
| P axes | 50 ± 26 | 53 ± 17 | 48 ± 32 | | 0.264 |
| QRS axes | 37 ± 43 | 31 ± 51 | 42 ± 35 | | 0.156 |
| T axes | 28 ± 33 | 20 ± 43 | 34 ± 21 | | 0.025 |
| **Signal-averaged ECG (SAECG)** | | | | | |
| Total QRS duration (filtered) (ms) | | 117 ± 26 | 128 ± 32 | 109 ± 19 | <0.001 |
| Duration of HFLA signals < 40 mv (ms) | | 40 ± 26 | 52 ± 30 | 31 ± 18 | <0.001 |
| RMS voltage in terminal 40 ms (mV) | | 34 ± 26 | 23 ± 18 | 42 ± 27 | <0.001 |
| Mean voltage in terminal 40 ms (mV) | | 24 ± 19 | 17 ± 15 | 29 ± 21 | <0.001 |

*Abbreviations: TWI:T wave inversion; RBB: right bundle branch block; BBB: bundle branch block; PVC: premature ventricular contractions; HFLA: high frequency low amplitude; RMS: root mean square.*

*.^a^* *SAECG was performed in 109 patients.*

*^b^ 24 Holter monitoring was performed in 50 patients.*

**eTable 3.** Imaging-derived characteristics of definite and non-definite ARVC

| **Parameters** | **Total, n=133** | **Definite**  **n = 60** | **Non-definite n=** **73** | **P value** |
| --- | --- | --- | --- | --- |
| **Echocardiography** |  |  |  |  |
| RA area (cm^2^) | 16 ± 7 | 19 ± 8 | 14 ± 4 | <0.001 |
| **RV data** |  |  |  |  |
| RVOT PLAX (cm) | 3.1 ± 0.8 | 3.5 ± 0.9 | 2.8 ± 0.6 | <0.001 |
| Proximal RVOT PSAX (cm) | 3.3 ± 0.7 | 3.6 ± 0.8 | 3.0 ± 0.6 | <0.001 |
| 4C base RV1(cm) | 3.7 ± 0.9 | 4.2 ± 1.0 | 3.4 ± 0.6 | <0.001 |
| 4C mid RV2 (cm) | 3.2 ± 0.8 | 3.5 ±0.9 | 2.9 ± 0.5 | <0.001 |
| 4C length RV3 (cm) | 7.1 ± 1.2 | 7.5 ± 1.3 | 6.8 ± 0.9 | <0.001 |
| RVEDA (cm^2^) | 22 ± 8 | 27 ± 10 | 18 ± 4 | <0.001 |
| RVESA (cm^2^) | 13 ± 7 | 17 ± 8 | 9.4 ± 2.5 | <0.001 |
| FAC (%) | 43 ± 11 | 37 ± 11 | 48 ± 6 | <0.001 |
| TAPSE (cm) | 2.1 ± 0.5 | 1.9 ± 0.5 | 2.2 ± 0.4 | 0.001 |
| **LV data** | | | | |
| EDV (ml) | 91 ± 29 | 89 ± 27 | 94 ± 32 | 0.408 |
| ESV (ml) | 37 ± 16 | 39 ± 16 | 35 ± 15 | 0.139 |
| EF (%) | 60 ± 10 | 56 ± 12 | 63 ± 7 | <0.001 |
| LA area (cm²) | 16 ± 4 | 17 ± 4 | 16 ± 3 | 0.172 |
| LA volume (ml) | 40 ± 15 | 42 ± 17 | 39 ± 13 | 0.269 |
| **CMR** | | | | |
| **RV characteristics** | | | | |
| RVEDV (ml) | 184 ± 57 | 195 ± 65 | 167 ± 39 | <0.001 |
| RVESV (ml) | 105 ± 57 | 125 ± 65 | 76 ± 21 | <0.001 |
| RV EF (%) | 45 ± 14 | 39 ± 15 | 54 ± 7 | <0.001 |
| **LV characteristics** | | | | |
| LVEDV (ml) | 155 ± 52 | 155 ± 60 | 155 ± 38 | 0.993 |
| LVESV (ml) | 66 ± 45 | 71 ± 55 | 58 ± 23 | 0.176 |
| LV EF (%) | 60 ± 14 | 58 ± 17 | 63 ± 8 | 0.095 |
| ^a^ LGE present, n (%) | 28 (45) | 24 (67) | 4 (15) | < 0.001 |
| LV LGE | 17 (27) | 14 (39) | 3 (12) | 0.022 |
| RV LGE | 24 (39) | 22 (61) | 2 (8) | < 0.001 |

*Abbreviations: RA area: right atrial area; RVOT PLAX: right ventricular outflow tract parasternal long axis; RVOT PSAX: right ventricular outflow tract parasternal short axis; 4C base RV1: 4 chamber right ventricular basal diameter; 4C mid RV2: 4 chamber right ventricular mid diameter; 4C length RV3: 4 chamber right ventricular length; RVEDA: right ventricular end diastolic area; RVESA: right ventricular end systolic area; RVFAC: right ventricular fractional area change; TAPSE: tricuspid annular plane systolic excursion; LVEDD: left ventricular end-diastolic volume; LVESV: left ventricular end-systolic volume; EF: ejection fraction; LA area: left atrial area; LA volume: left atrial volume; LGE: late gadolinium enhancement.*

*^a^ LGE was assessed in 62 patients only.*


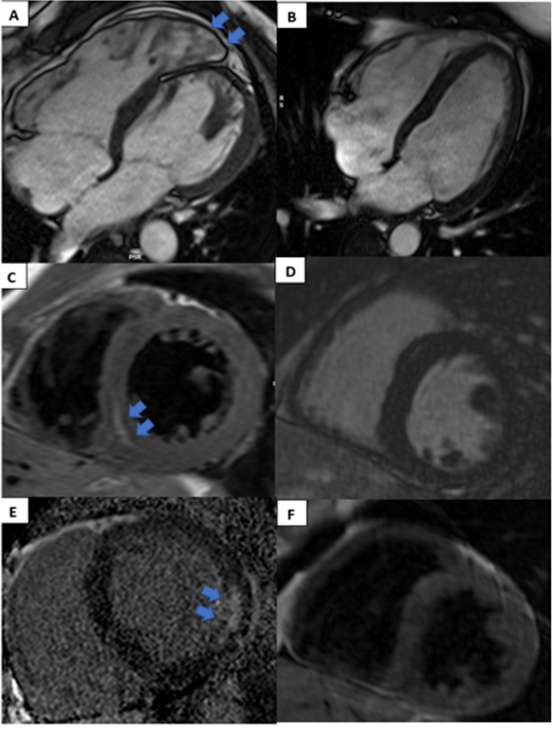


**eFigure 1.** (left) A definite stage ARVC, 41 year old endurance cyclist male. CMR shows (A) dilated and severely impaired right ventricle (RV) function, the apex is akinetic and the free wall has crenulated appearance (blue arrow). The Left ventricle (LV) is mildly dilated with mildly reduced function. (C and E) In the late phase after gadolinium contrast, the areas of high signal on T1w imaging are generally well matched with the areas of LGE with the exception of a subendocardial LGE focus in the mid lateral wall (blue arrow). (right) a non-definite ARVC, 39 year old male with a DSP pathogenic variant. CMR shows (B) Normal biventricular size and systolic function with no LGE (D and F).


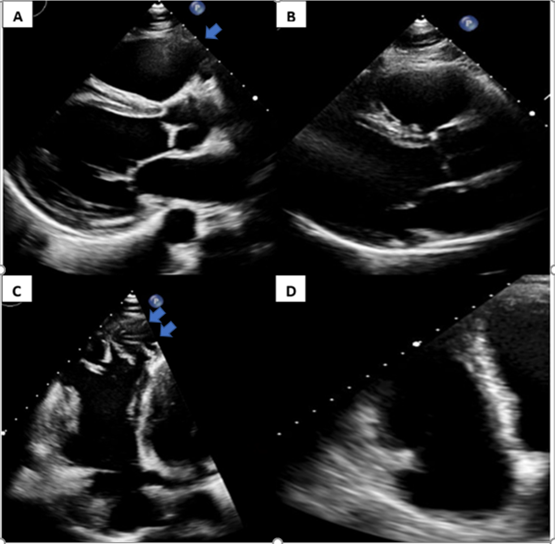


**eFigure 2.** (left) echocardiographic features of the same patient with definite diagnosis in parallel with the CMR findings from previous image. (A) Parasternal longs axis and (C) apical 4 chamber view showing dilated and trabeculated right ventricle (RV) (blue arrow), with thin and hypokinetic RV (mid to apex). (right) (B) Parasternal longs axis and (D) apical 4 chamber view showing normal biventricular size and function as demonstrated by CMR for the non- definite patient.


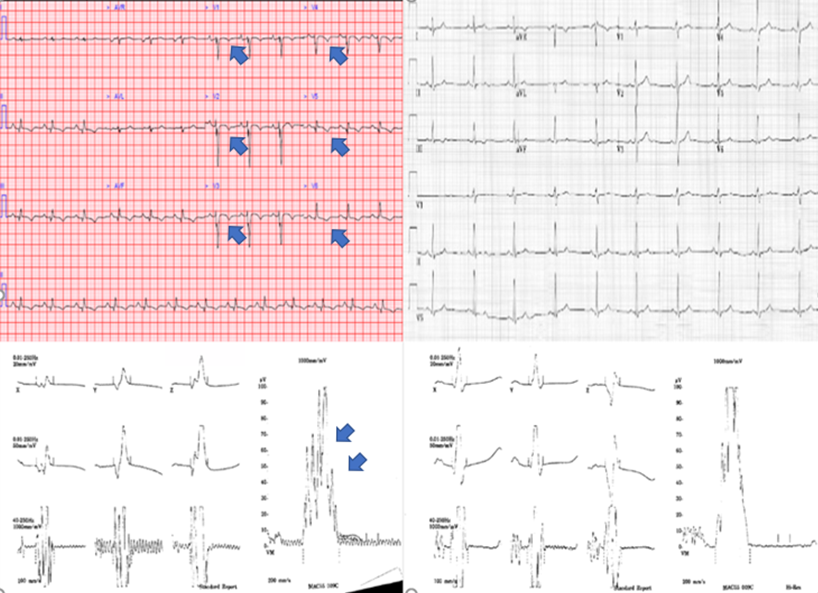


**eFigure 3.** Resting ECG of a definite ARVC patient (left up) showing T- wave inversion in V 1 to V6 (bule arrow) with (left down) signal average ECG showing Late potentials compared (blue arrow) to the non-definite patient with no T-wave inversion and no late potentials.
